# Exploring the manganese-dependent interaction between a transcription factor and its corresponding DNA: insights from gas-phase electrophoresis on a nES GEMMA instrument

**DOI:** 10.1007/s00216-024-05473-9

**Published:** 2024-08-22

**Authors:** Ivana Leščić Ašler, Katarina Radman, Zoe Jelić Matošević, Branimir Bertoša, Victor U. Weiss, Martina Marchetti-Deschmann

**Affiliations:** 1 Division of Physical Chemistry, Ruđer Bošković Institute, Zagreb, Croatia; 2 Department of Chemistry, Faculty of Science, University of Zagreb, Zagreb, Croatia; 3 Institute of Chemical Technologies and Analytics, TU Wien, Getreidemarkt 9-164 CTA, 1060 Vienna, Austria

**Keywords:** MntR transcription factors, Manganese metallosensors, Gas-phase electrophoresis, EMSA, nES GEMMA, DMA

## Abstract

Manganese ion homeostasis is vital for bacteria and is achieved via manganese-dependent transcription factors. Manganese mediation of transcription factor attachment to the corresponding oligonucleotide sequences can be investigated, e.g. via electrophoretic mobility shift assays (EMSA). Formation of specific biocomplexes leads to differences in the migration pattern upon gel electrophoresis. Focusing on electrophoresis in the gas-phase, applying a nano electrospray gas-phase electrophoretic mobility molecular analyzer (nES GEMMA) also known as nES differential mobility analyzer (nES DMA), and on transcription factors (MntR proteins) from *Bacillus subtilis* and *Mycobacterium tuberculosis*, we took interest in the gas-phase electrophoresis of the corresponding biospecific complexes. We compared nES GEMMA, separating analytes in the nanometer regime (a few to several hundred nm in diameter) in the gas-phase in their native state according to particle size, to EMSA data. Indeed we were able to demonstrate manganese-mediated attachment of MntR to target genomic sequences with both analytical techniques. Despite some inherent pitfalls of the nES GEMMA method like analyte/instrument surface interactions, we were able to detect the target complexes. Moreover, we were able to calculate the molecular weight (MW) of the obtained species by application of a correlation function based on nES GEMMA obtained data. As gas-phase electrophoresis also offers the possibility of offline hyphenation to orthogonal analysis techniques, we are confident that nES GEMMA measurements are not just complementary to EMSA, but will offer the possibility of further in-depth characterization of biocomplexes in the future.

**Graphical Abstract:**

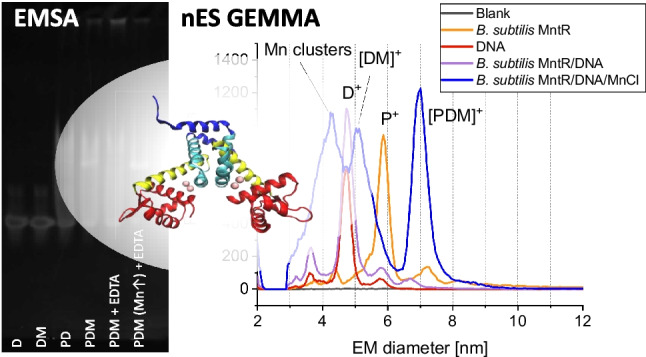

## Introduction

Manganese(II) ions are essential for a variety of cellular bacterial processes, such as DNA replication and resistance to oxidative stress. On the other hand, manganese excess is toxic [1]. In *Bacillus subtilis*, Mn^2+^ ion homeostasis is regulated by the transcription factor MntR. Binding of Mn^2+^ ions increases the DNA-binding affinity of *B. subtilis* MntR protein (BMntR), allowing it to regulate manganese import systems in a concentration-dependent way. MntR functions as a homodimer, with each monomer containing a dimerization domain and a DNA-­binding domain and a binuclear metal-­binding site [2]. It has been proposed that one of the bound metal ions is responsible for proper orientation of domains to enable DNA binding. In a previous study, we employed molecular dynamics (MD) simulations to investigate changes in *B. subtilis* MntR structure and dynamics occurring upon binding of Mn^2+^. Analyses of MD simulations indicated that binding of Mn^2+^ in one of the binding sites keeps protein domains in closer proximity [3].

Manganese-dependent complex formation can in principle be investigated by gel electrophoresis under native conditions. With such a setup, transcription factors binding to corresponding oligonucleotide sequences in the presence of Mn^2+^ travel differently than individual components through a polymeric sieving matrix by application of an electric field (electrophoretic mobility shift assay, EMSA). A deduction of molecular weight (MW) of sample components is difficult, as separations are based on charges and hydrodynamic size values of compounds alike. It is the aim of our manuscript to demonstrate that gas-phase electrophoresis on a nano electrospray gas-phase electrophoretic mobility molecular analyzer (nES GEMMA) is capable to size-separate obtained species and to yield molecular weight values of compounds of interest.

With nES GEMMA, analytes are electrosprayed from a volatile electrolyte solution via a cone-tipped capillary by application of pressure, an electric field, and a sheath flow of CO_2_ and particle-free, ambient air. Following droplet formation, the electrolyte is evaporated and at the same time charge equilibration occurs in a bipolar atmosphere induced by e.g. a ^210^Po alpha particle source, a soft X-ray charger, or a bipolar corona discharge process [4]. As a consequence, the majority of analyte molecules loses all charges, a certain percentage of analyte molecules is singly charged, and the percentage of two- or more-fold charged analyte particles is negligible [5]. Based on electrophoretic principles, the singly charged particles are then separated only according to the particle size in a tunable electric field and a high laminar sheath flow of particle-free, compressed air in a nano differential mobility analyzer (nDMA). Subsequently, monomobile particle fractions are introduced to an ultrafine condensation particle counter (CPC), where particles are counted upon passing of a focused laser beam after a nucleation step in a supersaturated atmosphere of n-butanol or water. As a consequence, obtained spectra give particle number concentrations in accordance with recommendations of the European Commission (2011/696/EU, October 18, 2011, updated version 2022/C 229/01, June 10, 2022) in relation to the electrophoretic mobility (EM) diameter of particles. The EM diameter describes the diameter of a spherical equivalent of the analyte in question and is related to the voltage setting necessary for particles to pass the nDMA size filter. Such a setup was first described by Kaufmann and colleagues in 1996 [6]. Since then, it has been known under several names; instead of “nES GEMMA,” also “MacroIMS,” “LiquiScan ES,” “SMPS,” or “nES DMA” are used for instrument description.

Besides proteins and protein aggregates as first described by Kaufmann et al. [6], also various other (bio-)nanoparticle materials starting from lipid aggregates (lipoproteins, liposomes, extracellular vesicles) [7–9], to viruses [10–13] and virus-like particles [14] as well as organic [15] and inorganic nanoparticles [16–18], carbohydrates [19], DNA [20], and polymeric samples [21] have been targeted by this analytical technique (only exemplary citations are given). Next to information on the size equivalent of analytes and their particle number concentrations, application of a corresponding correlation function allows to calculate the analytes MW values in good approximation based on their EM diameters, as first described by Bacher and colleagues for proteins [22] and as recently reviewed [23]. In addition, by setting the voltage of the nDMA to a constant value, particles of a given EM diameter can be size-collected on various sample support surfaces for further characterization based on orthogonal analytical techniques. For instance, atomic force microscopy (AFM) [24], mass spectrometry (MS) [25], or immunoanalytical techniques [26] allow thorough characterization of components even from complex samples. nES GEMMA unique capabilities from analyte MW determination to its offline hyphenation to orthogonal analysis techniques contribute to the importance of this approach in general and for the characterization of manganese-dependent transcription factors in the particular case.

Furthermore, nES GEMMA analyses can be seen as first proof-of-concept measurements for ESI-MS characterization of analytes, as both techniques, despite being different in many ways, share at least in part the process of analyte transfer from the liquid to the gas-phase.

Therefore, it was the aim of our investigation to demonstrate that gas-phase electrophoresis on a nES GEMMA instrumentation is also capable of yielding information on manganese-mediated complex formation between transcription factors and oligonucleotides. In doing so, we focused on BMntR as a transcription factor with a well-known oligonucleotide binding capacity and MntR from *Mycobacterium tuberculosis* (MMntR) as an additional component of interest [27, 28]. Previous nES GEMMA experiments had already successfully targeted complex formation based on antibody/antigen [29] or lectin/glycoprotein interactions [30]. To our knowledge, the metal-mediated binding of transcription factors to genomic material has never been shown via gas-phase electrophoresis to date.

## Materials and methods

### Chemicals

Ammonium acetate (≥ 99.99% trace metal basis), ammonium hydroxide (approx. 28–30% [w/v] ammonia in water, ACS reagent), lyophilized albumin from chicken egg white (≥ 98% via agarose gel electrophoresis), and manganese chloride tetrahydrate (≥ 99%) were obtained from Sigma-Aldrich (St. Louis, MO, USA). Water from a Millipore apparatus with 18.2 MΩcm resistivity at 25 °C (Merck, Darmstadt, Germany) was used.

### Instrumentation

Gas-phase electrophoresis was performed on a nES GEMMA instrument (TSI Inc, Shoreview, MN, USA) consisting of a 3480C nES aerosol generator applying in-house grinded cone-tipped fused silica capillaries (25-µm inner diameter) [31] and a bipolar corona discharge process (MSP 1090) [32], a 3080C classifier with a 3085 nDMA, and a 3776C n-butanol-driven ultrafine condensation particle counter. MacroIMS manager v2.0.1 was used as instrument software. Particle-free air was dried prior to application (Donaldson Variodry Membrane Dryer Superplus obtained via R. Ludvik Industriegeräte, Vienna, Austria).

### Protein preparation and purification

MntR protein from *B. subtilis* (BMntR, UniProt no. P54512) was purified as before [3]. In its final preparation in 20 mM HEPES buffer pH 7.2 containing 200 mM NaCl and 10% [v/v] glycerol, the protein concentration measured 0.7 mg/mL (per monomer). Aliquots were stored at − 80 °C until use.

MntR protein from *Mycobacterium tuberculosis* (MMntR, UniProt no. A0A045JFF4) was purified essentially by the same procedure as BMntR. In brief, the plasmid N-His_MmntR_pET-45b( +) containing the gene for *M. tuberculosis* MntR protein, with a sequence coding for 6 His and the recognition site for HRV-3C protease added at its N- terminal end, was purchased from GenScript Biotech (Leiden, the Netherlands). The protein was overexpressed in *Escherichia coli* cell strain BL21-CodonPlus(DE3)-RIL (Agilent Biotechnology, Santa Clara, CA, USA), after induction by 0.5 mM IPTG (isopropyl β-d-1-thiogalactopyranoside). Protein purification was performed at 4 °C. Cells were lysed by a high-pressure homogenizer (Avestin Emulsiflex C3, Avestin Inc., Ottawa, ON, Canada) and the protein extract was loaded onto a column of Ni–NTA agarose (Protino, Macherey–Nagel, Düren, Germany). After elution of bound proteins with 0.3 mM imidazole, His-tags were cleaved off with HRV-3C protease (Thermo Fisher Scientific, Waltham, MA, USA) during an overnight dialysis. For additional purification of the protein, size-exclusion chromatography was performed via a Superdex 75 pg HiLoad 16/600 column (Cytiva Life Sciences, Marlborough, MA, USA) on the ÄKTA Pure FPLC system (Cytiva Life Sciences). The buffer was the same as for BMntR, except for the pH, which was 8.0 for MMntR instead of 7.2 as for BMntR, and the sodium chloride content. The final sample of purified protein (2 mg/mL per monomer, in 20 mM HEPES buffer pH 8.0 containing 100 mM NaCl and 10% [v/v] of glycerol) was stored in aliquots at − 80 °C until use.

The purification of proteins was monitored by electrophoresis under denaturing conditions (SDS-PAGE) and protein concentration was determined utilizing protein’s molar extinction coefficients at 280 nm of 18,910 M^−1^ cm^−1^ for BMntR and 33,460 M^−1^ cm^−1^ for MMntR (as calculated using the ProtParam tool at ExPasy.org).

### DNA oligomers

The double-stranded DNA oligonucleotide containing *mntH* recognition sequence in *B. subtilis* (5′-AAAATAATTTGCCTTAAGGAAACTCTTT-3′, [33]) and two oligonucleotides containing sequences of genes that were validated as targets of MntR in *M. tuberculosis* (Rv2058c: 5′-CATTATTGCGACGTCGACGGTACAGTGCCA-3′, Rv1283c: 5′-ACTCAGATCGACGCCGCGGTGAATTTTAAC-3′, [28]) were purchased from Macrogen Europe (Amsterdam, the Netherlands) and dissolved in doubly distilled water giving stock solution of 100 µM. These stock solutions were diluted to a desired concentration for GEMMA measurement in 40 mM ammonium acetate, pH 8.4.

### Sample preparation

Thawed aliquots of MntR protein samples were transferred to 40 mM ammonium acetate, pH 8.4, via successive rounds of spin filtration in centrifugal units (modified PES, 10 kDa molecular weight cutoff, 500 μL, VWR International, Radnor, PA, USA). Three rounds of dilution/concentration were performed, each round diluting 10–30 μL of protein to 500 μL total volume with 40 mM ammonium acetate, pH 8.4. The protein concentration was measured after desalting, utilizing the molar extinction coefficients at 280 nm of each protein. Standard proteins were prepared in 40 mM ammonium acetate, pH 8.4, to desired concentrations. Typically, protein and DNA concentrations were in the single-digit µM range for nES GEMMA analyses. In addition, 250 µM MnCl_2_ was applied, where indicated.

### Electrophoretic mobility shift assay (EMSA)

The assay was performed essentially as in [34], in 20 μL reaction volumes each containing 50 µg/mL BSA (bovine serum albumin), 50 mM NaCl, 1 mM DTT (dithiothreitol), and 10% glycerol in 10 mM Tris–HCl buffer pH 7.6. Stock solutions of Mn^2+^ (MnCl_2_) were prepared fresh before use and added to reaction mixtures to the final concentration of 4 mM (or 16 mM in reaction with 8 mM EDTA). DNA (stock solutions prepared for GEMMA measurements were used) was added to the concentration of 2.5 μM. MntR proteins were added to the concentration of 90 µM, thus ensuring complete binding of DNA to the protein. The reactions were incubated at room temperature for 15 min. The samples were dyed with bromophenol blue and 5 μL of each sample was loaded on an 8% polyacrylamide gel (casted in-house: 8% w/v polyacrylamide, 5% v/v glycerol, 0.075% w/v ammonium persulfate, 0.075% v/v TEMED, in TB buffer (89 mM Tris, 89 mM borate), pH 7.6). The gel was equilibrated prior to sample loading at 100 V and 4 °C for 30 min and the samples were separated at 100 V and 4 °C for 60 min, in TB buffer, pH 7.6. DNA was visualized in the gel by staining with ethidium bromide.

### Nano electrospray gas-phase electrophoretic mobility molecular analyzer (*nES GEMMA*)

The nES was operated under the following conditions: 0.1 L per minute (Lpm) CO_2_, 1.0 Lpm compressed, particle-free air, 4.0 pounds per square inch differential (psid) pressure in the sample chamber and an applied voltage of approx. 1.8 kV yielding a stable Taylor cone and currents in the range of − 350 nA. The sheath flow in the nano DMA was set to 15 Lpm. Voltage scanning was for 115 s between 10 V and 10 kV; 5 s was applied for retracing the voltage to initial conditions. Ten voltage scans were combined via their median to yield a corresponding nES GEMMA spectrum. Capillaries were exchanged after measurements with MnCl_2_ and when switching to a different protein. A corresponding EM diameter/MW correlation was setup as described elsewhere [19].

## Results and discussion

The objective of our investigation was to showcase the ability of gas-phase electrophoresis on a nES GEMMA instrumentation to monitor the complex formation between manganese-dependent transcription factors and their corresponding genomic material. Besides the opportunities given by the selected analytical technique, we will also focus on pitfalls of gas-phase electrophoresis of such complex samples.

### BMntR protein binds to oligonucleotides in a manganese-dependent manner

**Fig. 1:**
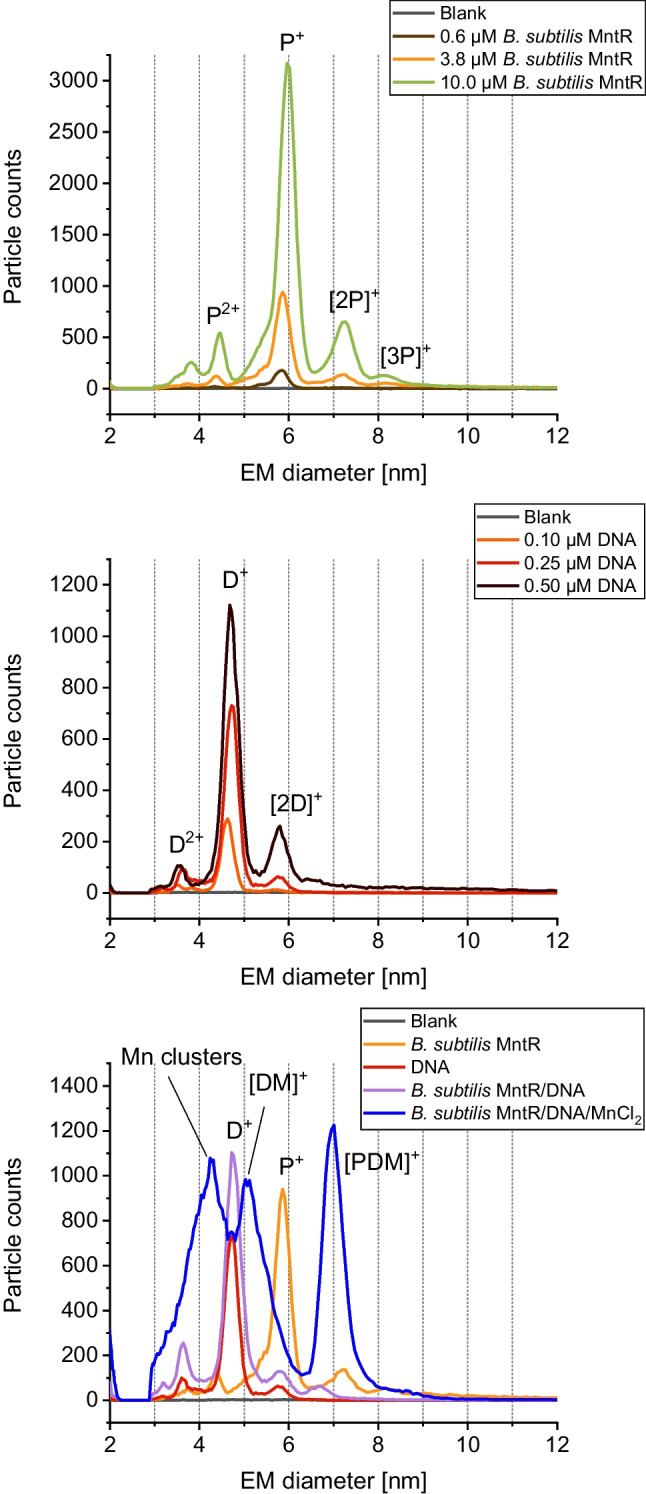
nES GEMMA results for analysis of MntR from *B. subtilis* (upper panel), a corresponding oligonucleotide (middle panel), and mixtures of binding partners (lower panel). Gas-phase electrophoresis of individual compounds shows distinct peaks for protein monomers (P^+^) as well as some concentration-dependent multimerization ([2P]^+^, [3P]^+^). To a certain extent, double-charged species (P^2+^) are detected. Similar results were obtained for analysis of DNA oligomers (D^+^, D^2+^, [2D]^+^). Mixing of both components yields a distinct peak for the biospecific complex only under manganese ion mediation ([PDM]^+^). For the lower panel, 1.3 µM BMntR, 0.25 µM DNA, and 250 µM MnCl_2_ were used in respective samples

As demonstrated in a previous study [2], MntR from *B. subtilis* (BMntR) binds to a corresponding short DNA fragment in a manganese-dependent manner. Indeed, applying gas-phase electrophoresis on a nES GEMMA instrumentation, we were able to reproduce this finding (Fig. 1). In order to demonstrate binding, we analyzed both complexation partners—BMntR (P, upper panel) and the DNA oligonucleotide (D, middle panel)—at different concentrations prior to measurement of mixed samples (lower panel).

In detail, nES GEMMA relies on the application of an ES process to transfer non-volatile analytes from a liquid sample to the gas-phase. Depending on the instrument’s settings, a certain number of ES droplets is formed. If the number of analyte molecules exceeds the number of ES droplets, spray-induced multimerization of analytes can be observed. Only below a certain threshold, this effect can be circumvented [19]. In order to exclude unspecific, ES-based particle aggregation, we measured BMntR (Fig. 1, upper panel) and the applied DNA oligomer (Fig. 1, middle panel) at different concentrations. During these measurements, we detected native BMntR—in the case of MntR, the functional entity is a homodimer—at 5.9 ± 0.1 nm (*n* = 5) EM diameter (P^+^ species). For samples of higher concentration, dimers ([2P]^+^) or even trimers ([3P]^+^) of native protein assemblies were detected. Due to aforementioned reasons, these latter moieties most probably reflect spray-induced aggregates. In contrast, the applied DNA oligomer showed a peak at 4.7 ± 0.1 nm (*n* = 4) EM diameter (D^+^ species) also with spray-induced dimers when analyzing highly concentrated samples. To conclude, both species—protein and DNA—were discernible in terms of their EM diameter upon gas-phase electrophoresis. Furthermore, by choosing low analyte concentrations, it was the aim of our further experiments to reduce unspecific ES-based analyte aggregation to a possible minimum.

Following our initial experiments, we intended to demonstrate manganese-dependent formation of a protein/DNA complex ([PDM]^+^). In doing so, we mixed both binding partners either in the presence or absence of manganese ions and recorded corresponding spectra (Fig. 1, lower panel). Only in the presence of manganese mediation we observed a complex peak at 7.0 ± 0.1 nm EM diameter (*n* = 3); in the absence of manganese ions, no such binding was observed. It is of note that (i) due to the EM diameter of the complex peak as well as due to (ii) the recorded particle count values and due to (iii) the absence of the complex peak for the PD mixture in the absence of manganese ions, we are confident to indeed detect the specific PDM complex at 7.0 nm EM diameter and no unspecific aggregation between P and D species.

In a next step, we subjected all observed peaks to molecular weight (MW) determination by application of a corresponding correlation function between particle EM diameter and MW values [19]. Due to updates of several nES GEMMA instrument parts since our publication in 2018 [19] and hence changed instrumental geometries, a slight deviation from the published EM diameter/MW correlation soon became evident. Although obtaining a good approximation with the 2018 correlation, we conducted additional measurements on a number of standard proteins, including carbonic anhydrase, ovalbumin, bovine serum albumin, as well as beta-galactosidase, and subsequently recalculated the associated correlation in order to enhance the precision of calculated MWs for the present manuscript. Our new correlation valued MW [kDa] = 0.1185 ∙ EMD [nm] ^3.2078^. Via this updated correlation, we obtained a MW of 34.6 kDa for native BMntR at 5.9 nm EM diameter, 17.4 kDa for the oligonucleotide, and 60.5 kDa for the manganese-mediated complex at 7.0 nm EM diameter. Calculated values are thus in good accordance with theoretical data: 33.8 kDa for the MntR dimer, 17.2 kDa for the oligonucleotide, and 51.0 kDa for the manganese-mediated complex. It is of note that EM diameter to MW correlation functions for proteins and DNA are very similar enabling the application of a protein-based correlation in both cases. For the manganese-mediated complex, probably only a very approximate value is obtained (overestimation of MW values based on nES GEMMA data as reflected by presented numbers) as unspecific attachment of manganese ions leads to an increase in EM diameter which, however, is not based on an increased protein complex size but is nevertheless taken into account upon calculation.

### Pitfalls of *nES GEMMA* measurements of manganese-mediated complex formation

Transfer of analyte material in nES GEMMA is based on a nES process. Analytes are electrosprayed from a cone-tipped fused silica capillary. As most silanol groups on the capillary surface are deprotonated under basic pH conditions of the applied ammonium acetate electrolyte solution (pH 8.4 was used in our experiments), we ran into serious problems for measurement of some of our sample components, especially positively charged manganese ions and manganese containing complexes. However, we refrained from the application of an acidic electrolyte in order not to unfold the applied proteins.

**Fig. 2:**
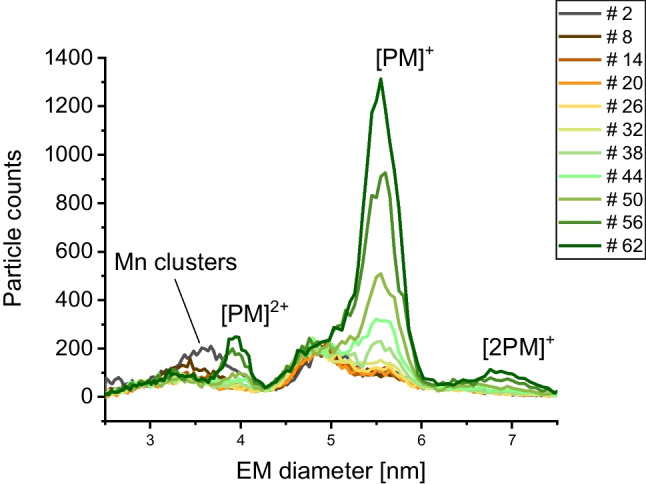
Gas-phase electrophoresis of a MntR sample from *B. subtilis* mixed with manganese ions. With increasing analysis time, the peak recorded for manganese clusters at low EM diameter values diminishes. At the same time, the peak assigned to BMntR ([PM]^+^) slowly increases. Both effects indicate analyte/instrument surface interactions at the pH of the applied electrolyte solution. Individual spectra of a measurement series are plotted

As a result, the time allowed for samples to pass through the nES capillary was significantly increased, especially for those containing proteins and manganese. As demonstrated in Fig. 2 for an applied protein/manganese (PM) sample, differences in the time for reaching stable electrospray conditions were in the order of several tens of minutes. It is of note that in Fig. 2, unlike for the other presented nES GEMMA–related figures, we depict individual scans from gas-phase electrophoresis.

Usually, we start recording spectra of a given sample after several minutes of equilibration time (typically 3–5 min). In this way, stable results can be obtained. After recording of several individual spectra, these are combined via their median in order to achieve removal of possible spikes in datasets. In contrast, PM samples yield very low particle count values for the protein/manganese complex. Electrospraying the PM sample for a longer time—considering that each EM diameter scan lasts for 115 s to ramp the DMA voltage and 5 s to adjust the voltage back to the initial value—reveals that the PM peak only very slowly increases in particle counts. It is just after roughly 120 min of continuously electrospraying the same sample that we obtained a stable signal for the manganese-mediated complex and recording of a corresponding dataset could be started. As a result, complex formation might be wrongly excluded in case the time of instrument equilibration and analysis is set too short.

Likewise, application of manganese ions within a sample can cause problems for the applied nES GEMMA instrumentation and probably as well for other nES- or ES-based analysis methods. During our measurements, we visually observed manganese depletion on the platinum electrode submerged into the sample. This effect can also be seen in corresponding spectra—over time the recorded number of Mn^2+^-based clusters is decreasing as observed from particle numbers (Fig. 2). Based on this visual observation, the attachment of manganese ions to the fused silica material of the capillary can also be safely assumed. Consequently, we highly recommend frequent exchange of the fused silica capillary as well as thorough cleaning of the electrode in order not to contaminate nominally manganese-free samples with material detached from surfaces. This, together with sufficiently long equilibration times of the instrumentation to new samples, will yield interpretable datasets.

### Application of gas-phase electrophoresis in the research of manganese-dependent transcription factors

**Fig. 3:**
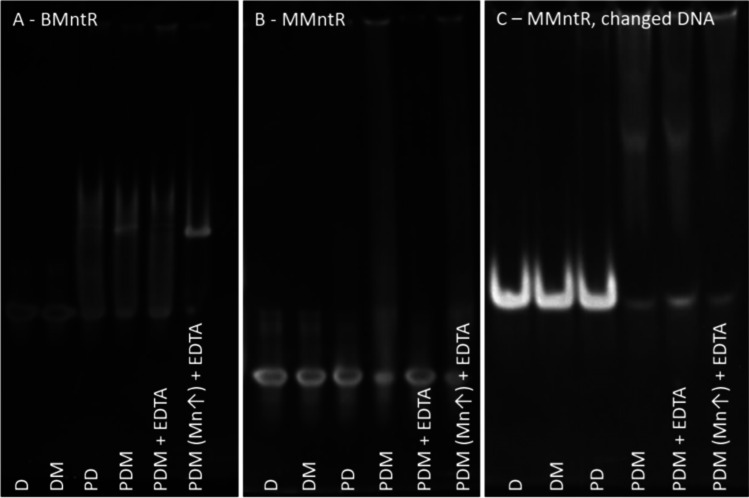
EMSA results for BMntR and MMntR proteins upon complexation to DNA oligonucleotides in the presence and absence of manganese ions. Protein interaction to oligonucleotides results in a shift of band migration to higher gel positions. Interestingly, BMntR also shows slight interaction to DNA in the absence of manganese ions (**A**). However, the presence of manganese ions results in more defined bands. More ambiguous results are observed for MMntR. Whereas no binding to the *mntH* promoter sequence oligonucleotide was found (**B**), complexation to the ABC transporter promoter sequence oligonucleotides was seemingly detected (**C**). However, corresponding results were vague

After demonstrating that gas-phase electrophoresis on a nES GEMMA instrumentation is a suitable method to follow the manganese-mediated binding of transcription factors to corresponding oligonucleotide sequences, we focused on another target protein. In doing so, we opted for MntR from *Mycobacterium tuberculosis* (MMntR) as the molecule of interest [27, 28]. As binding of MMntR to DNA was reported, we asked ourselves if slight modifications of the DNA sequence are already sufficient to influence binding of the transcription factor to its target. Thus, we chose two oligonucleotide sequences, with different deviations from the MMntR consensus binding sequence [28]. These sequences correspond to promoter regions for *mntH* (Rv2058c) and ABC transporter gene (Rv1283c), both involved in manganese transport in cells. In contrast to BMntR which exhibited binding to its target DNA molecule (Fig. 3A), MMntR failed to yield a defined, specific complex band via EMSA upon application of binding conditions to the *mntH* promoter (Fig. 3B) or to the oligonucleotide extracted from the promoter region of the ABC transporter gene (Fig. 3C). It seems that changes at the beginning of DNA sequence (as in Rv2058c) as well as spread out changes (as in Rv1283c), in comparison to the consensus sequence [28] both diminish affinity of MMntR-DNA binding. However, especially for the latter, EMSA results were not unambiguous.

**Fig. 4:**
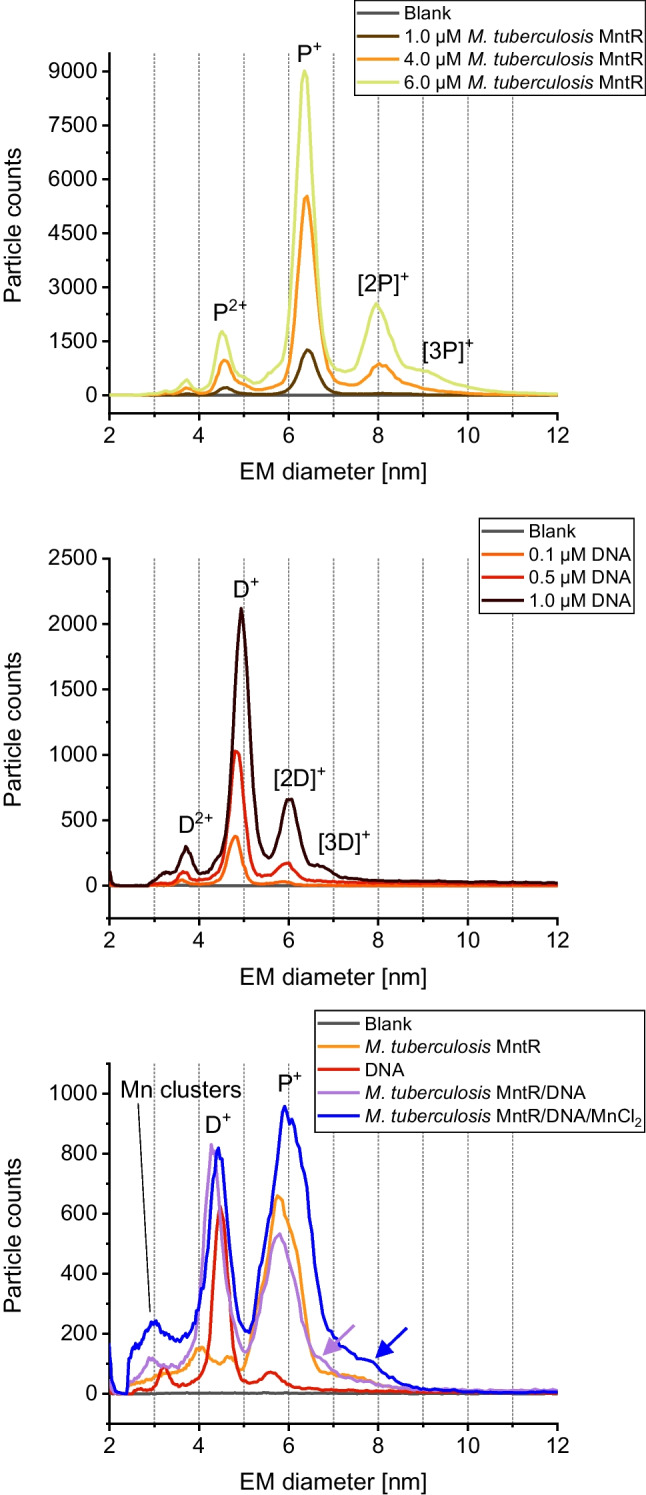
nES GEMMA results for binding of MntR from *M. tuberculosis* to the *mntH* promoter sequence oligonucleotide. Gas-phase electrophoresis shows distinct peaks for the protein (upper panel) and the DNA oligonucleotide alone (middle panel). In contrast to BMntR and along EMSA results, no complexation between MMntR and the chosen DNA sequence was detected, neither in the absence or presence of manganese ions (lower panel). A slight shoulder at the P^+^ or [PM]^+^ peak (indicated by arrows) probably results from unspecific complexation. For the lower panel, 0.7 µM MMntR, 0.25 µM DNA, and 250 µM MnCl_2_ were used in respective samples

Based on these experiments, we also investigated manganese-mediated MMntR binding to the *mntH* promoter sequence as well as the ABC transporter gene oligonucleotide via gas-phase electrophoresis to compare both methods—nES GEMMA and EMSA. As shown in Fig. 4, upper panel, for MMntR and Fig. 4, middle panel, for the corresponding *mntH* promoter sequence oligonucleotide, we could exclude unspecific aggregation of molecules to species of higher EM diameter in case analytes were applied in sufficiently low concentrations. In fact, MMntR showed a P^+^ peak at 6.3 ± 0.2 nm (*n* = 13) EM diameter, while the peak of the applied DNA D^+^ was recorded at 4.7 ± 0.2 nm (*n* = 5). Neither in the presence nor absence of manganese ions (Fig. 4, lower panel) a complex peak was found when mixing both complexation partners (a corresponding peak would have been expected at approx. 7.7 nm EM diameter based on the MW of complex partners analogous to previously described BMntR experiments). This indicates comparable results for nES GEMMA and EMSA experiments. It is of note that peak broadening of P^+^ and D^+^ species as well as slight shifts to higher EM diameters result from unspecific attachment of manganese to protein and DNA analytes upon drying of nES droplets, respectively [35].

**Fig. 5:**
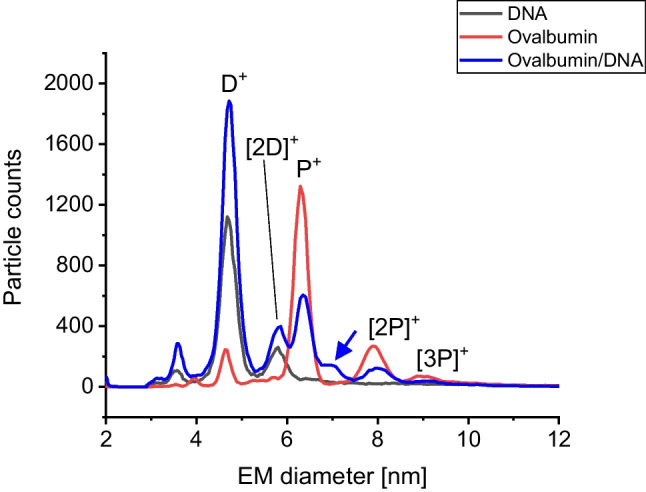
Gas-phase electrophoresis of ovalbumin as control protein either present with or without DNA in samples. nES GEMMA reveals slight unspecific protein/oligonucleotide interactions (indicated by an arrow)

In the case of mixed samples in the absence as well as in the presence of Mn^2+^, a slight shoulder at higher EM diameter values was recorded for MMntR (indicated by arrows). We concluded that these peaks observed as shoulders in spectra correlate to species resulting from some unspecific aggregation events most probably occurring during the nES process. To support this latter claim, we carried out a control experiment mixing ovalbumin with DNA again in the absence as well as in the presence of manganese ions (Fig. 5). It is of note that we opted for ovalbumin due to its similar MW (44.34 kDa) [19] and EM diameter (6.4 nm) when compared to MMntR. Also, in these experiments, we found a corresponding peak shoulder in spectra indicating unspecific aggregation between the protein and the oligonucleotide (indicated by an arrow). As a consequence, we reason that only when a correct three-dimensional complex structure had already formed in liquid phase (e.g., manganese-mediated between a transcription factor and a corresponding oligonucleotide), peaks of higher EM diameter are observed.

In the case of spray-induced multimerization, formed aggregates are smaller indicating non-defined particle attachment. It is of note that we found a similar effect in 2021 upon online coupling of a SEC instrumentation to a next-generation nES GEMMA [36]: Spray-induced dimers were lower in observed EM diameter than those aggregates, which were already present in liquid phase prior to the nES process, probably due to a lesser degree of order in nES-based aggregates. In case of manganese-dependent transcription factors and oligonucleotides, or respective control proteins and oligonucleotides, this effect seems to be even more pronounced.

Results obtained for the ABC transporter gene–based oligonucleotide (data not shown) were comparable to data based on the *mntH* promoter sequence. Hence, it is evident that nES GEMMA enables in some cases easier data evaluation upon investigation of complex formation than when focusing solely on EMSA experiments.

## Conclusion

Focusing on transcription factors and corresponding oligonucleotide sequences, we followed up on our hypothesis that gas-phase electrophoresis on a nES GEMMA instrumentation is capable of demonstrating manganese-mediated complex formation. Indeed, within the scope of our project, we succeeded to demonstrate its capability to investigate biospecific binding of compounds. In addition, nES GEMMA complements classical EMSA by additional data regarding the studied systems, providing their deeper understanding.

Furthermore, besides the confirmation of specific binding between sample components, gas-phase electrophoresis offers the possibility of MW determination by application of a corresponding correlation function [19]. In addition, offline hyphenation of the technique to orthogonal analysis methods enables in-depth analyte characterization as demonstrated for other analytes in already published studies. Therefore, we conclude that gas-phase electrophoresis, despite some method inherent pitfalls, which we discussed likewise in our manuscript, like analyte/instrument surface interactions, is capable to yield important information in the field of biological complex formation. As demonstrated, unspecific particle aggregation was negligible in the investigated cases.
